# A Vibration-Based MEMS Piezoelectric Energy Harvester and Power Conditioning Circuit

**DOI:** 10.3390/s140203323

**Published:** 2014-02-19

**Authors:** Hua Yu, Jielin Zhou, Licheng Deng, Zhiyu Wen

**Affiliations:** 1 College of Optoelectronic Engineering, Chongqing University, Chongqing 400044, China; E-Mails: 20120802065@cqu.edu.cn (J.Z.); dlc@cqu.edu.cn (L.D); wzy@cqu.edu.cn (Z.W.); 2 Key Laboratory for Optoelectronic Technology & Systems, Ministry of Education of China, Chongqing 400044, China; 3 National Key Laboratory of Fundamental Science of Micro/Nano-Device and System Technology, Chongqing 400044, China

**Keywords:** energy harvester, PZT, MEMS, power conditioning circuit

## Abstract

This paper presents a micro-electro-mechanical system (MEMS) piezoelectric power generator array for vibration energy harvesting. A complete design flow of the vibration-based energy harvester using the finite element method (FEM) is proposed. The modal analysis is selected to calculate the resonant frequency of the harvester, and harmonic analysis is performed to investigate the influence of the geometric parameters on the output voltage. Based on simulation results, a MEMS *Pb*(*Zr*,*Ti*)*O_3_* (PZT) cantilever array with an integrated large *Si* proof mass is designed and fabricated to improve output voltage and power. Test results show that the fabricated generator, with five cantilever beams (with unit dimensions of about 3 × 2.4 × 0.05 mm^3^) and an individual integrated Si mass dimension of about 8 × 12.4 × 0.5 mm^3^, produces a output power of 66.75 μW, or a power density of 5.19 μW**·**mm^−3^**·**g^−2^ with an optimal resistive load of 220 kΩ from 5 m/s^2^ vibration acceleration at its resonant frequency of 234.5 Hz. In view of high internal impedance characteristic of the PZT generator, an efficient autonomous power conditioning circuit, with the function of impedance matching, energy storage and voltage regulation, is then presented, finding that the efficiency of the energy storage is greatly improved and up to 64.95%. The proposed self-supplied energy generator with power conditioning circuit could provide a very promising complete power supply solution for wireless sensor node loads.

## 1. Introduction

The use of wireless sensors and implanted medical electronic systems has developed rapidly in recent years. They are usually powered by standard batteries which become depleted within a relatively short timeframe, but the replacement or recharge of batteries is a major bottleneck for wide deployment of wireless sensor nodes (WSN). Moreover, while the size of electronic circuitry has shrunk thanks to the advent of integrated circuit technology, batteries are nowadays often the most bulky devices in wireless sensor nodes. Vibration-based MEMS power generators present an amazing solution to supply power for wireless sensor nodes, which can generate mW or μW level power. Vibration energy harvesting has been exploited for converting ambient kinetic energy into electric energy by several different transduction mechanisms, including piezoelectric, electromagnetic and electrostatic methods. Among these three energy harvesters, the piezoelectric energy harvester (PEH) has a high electromechanical coupling effect, and requires no external voltage sources, it is compatible with MEMS technology, and accordingly has received much recent attention [1–4].

The piezoelectric material chosen for this research is PZT, because it is a well-known and well-characterized material with a high piezoelectric coefficient. Vibration energy harvesters must be able to respond to the low frequency and low acceleration vibrations that usually exist in the environment. Moreover, the energy harvester should generate as much energy as possible in order to supply enough power for the follow-up loads. Recently, MEMS technology has been applied toward the development of energy harvesters, and many piezoelectric MEMS energy harvesters have been developed. Renaud *et al.* proposed a fabricated MEMS-based PZT cantilever micro-generator with an integrated proof mass that can generate an average power of 40 μW at 1.8 kHz vibration frequency [5]. Jeon *et al.* developed a *d33* mode thin film PZT power generating device with interdigitated electrodes that can generate an average power of 1.0 μW from 108 m/s^2^ vibration acceleration at its resonant frequency of 13.9 kHz [6]. However, the above two energy harvesters' resonant frequencies are very high. Fang *et al.* fabricated a MEMS-based PZT cantilever power generator with a nonintegrated Ni proof mass that can generate 2.16 μW from 10 m/s^2^ vibration acceleration at its resonant frequency of 609 Hz. The nickel metal mass on the tip of the cantilever is used to decrease the structure's resonant frequency for the application under low-frequency vibration, but it cannot to be micro-machined by MEMS technology [7]. Similar to the previous work, Liu *et al.* used the previous cantilever structure to construct a power generator array to improve power output and frequency flexibility. Although they demonstrated that power density was high, the proof mass was not also integrated with the cantilever which will be an additional difficulty in production [8]. Muralt *et al.* designed and fabricated a micro power generator of thin film PZT laminated cantilever with proof mass and interdigitated electrodes which could generate a voltage of 1.6 V and power of 1.4 μW when excited under 20 m/s^2^ vibration acceleration at 870 Hz resonant frequency [9]. Over the past two years, some new structures or new piezoelectric materials have been applied in energy harvesters, which attain lower resonant frequency and more output power, but the power conditioning circuit for the improvement of high internal output impedance about piezoelectric energy harvester has rarely been discussed in these references [10–12]. From these previous studies, at least three conclusions can be reached and summarized as follows: Firstly, most MEMS vibration energy harvesters usually work under high frequency environment conditions, which limits the scope of application for these devices, because the frequencies of available ambient vibration sources are relatively low. Secondly, the delivered power output of those MEMS micro-generators is relatively low, so the architecture and parameters of energy harvester must be optimized as far as possible in order to increase output power. Thirdly, a power conditioning circuit is absolutely necessary for piezoelectric energy harvesters in order to extract the maximum power from the harvester, store energy and then regulate the voltage for meeting the demands of the loads, such as wireless sensor nodes [13].

In this paper, a complete design flow of a self-powered MEMS PZT vibration energy harvesting system for wireless sensor nodes is presented, which includes the design method of how to increase the output power and voltage of the energy harvester. A design method of low resonant frequency for the vibration energy harvester is also verified. This paper especially gives the theoretical calculation formula of the resonant frequency, the simulation value using ANSYS FEM software, and the experimental test results. It's shown that their relative errors are very small, which provided a reference design method of the resonant frequency for the vibrational energy harvester. Moreover, the total output power and voltage are greatly improved by improving the PZT film material preparation technology and MEMS cantilever array fabrication process, which lays a strong foundation of the latter power condition circuit. Finally, in order to improve the high internal impedance characteristics of the PZT generator, we design an efficient autonomous power conditioning circuit, with the functions of impedance matching, energy storage and voltage regulation, finding that the efficiency of the energy storage is greatly improved. The proposed whole energy harvesting system could provide a complete self-powered supply solution to wireless sensor node loads, which is regarded as a reference method for implementing a battery-less wireless sensor network.

## 2. Design of the MEMS Piezoelectric Energy Harvester

### 2.1. Architecture Design

**Figure 1.**
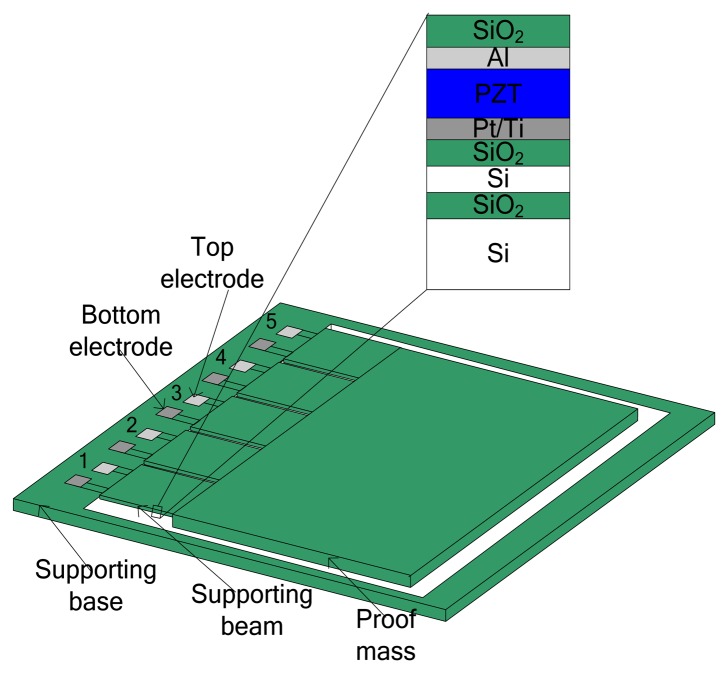
Cross section structure of MEMS piezoelectric cantilever array.

The cross-section structure of the MEMS piezoelectric cantilever array is shown in Figure 1. The cantilever array device is designed to resonate at a specific low vibration frequency by using a large integrated Si proof mass. The bending results in a strain distributed along the beam, which is then converted to electrical energy through the transverse mode (*d31*) piezoelectric effect. The Pt/Ti and Al electrodes are patterned in order to generate strain in parallel to the electric field, which forms the *d31* mode of the piezoelectric element. When an input vibration applies acceleration to the beam structure, the effective mass converts the input acceleration into force. The relative displacement causes the PZT layer to be tensed or compressed, which in turn induces a charge shift and accumulation due to the piezoelectric effect. Electrodes collect the generated charge and electrical damping results. The magnitude of the electric charge voltage is proportional to the stress induced by the relative displacement. Material properties and structural parameters of the piezoelectric energy harvester are listed in Table 1.

### 2.2. Output Power

Mechanical power is converted into electrical power when damping is present. For a sinusoidal excitation vibration, the electrical power generated by the system is given in Equation (1) [14]:

(1) $$P=\frac{m\zeta Y^{2}\left(\frac{\omega }{\omega_{n}}\right)\omega^{3}}{{\left[1-{\left(\frac{\omega }{\omega_{n}}\right)}^{2}\right]}^{2}+{\left[2\zeta \frac{\omega }{\omega_{n}}\right]}^{2}}$$

where *ζ* is the damping ration (*ζ* = *q*/*2mω_n_*); *ω_n_* and *Y* is the resonant frequency and the amplitude of vibration respectively, and *ω* is the excitation frequency. It's obvious that the maximum output power occurs at the resonant frequency of the generator if *ω* is equal to *ω_n_* for the system without damping:

(2) $$P_{\text{max}}=P\left(\omega_{n}\right)=\frac{mY^{2}{\omega_{n}}^{3}}{4\zeta }$$

It also can be concluded from Equation (2) that the value of the maximum power is indeed finite, and reduction of the damping factor results in increased mass displacement, which is ultimately limited by the size and geometry of the device. Equation (3) also expresses the power in terms of the excitation acceleration magnitude of the input vibration:

(3) $$P_{\text{max}}=\frac{mA^{2}}{4\zeta \omega_{n}}$$

where *A* is excitation acceleration magnitude, and its formula is $A=\omega_{n}^{2}Y$. Several conclusions may be drawn from the above equations. Firstly, if the resonant frequency of the generator is set equal to the proof mass frequency, the maximum power generated is limited by the movement of the proof mass. Thus, the maximum power can be expressed in terms of maximum distance the proof mass can move. Secondly, for applications where the frequencies of vibration are well defined and concentrated around one point, a low damping factor will provide higher peak responses and power generation. Thirdly, it can also be seen that power is proportional to the proof mass, so a large proof mass is always desirable for energy harvesting. Finally, the harvester's damping *ζ* is composed of the mechanical damping *ζ_m_* and electrical damping *ζ_e_*. The maximum electrical output power is equal to half of the value in Equation (2) when the electrical damping matches the mechanical damping [15].

### 2.3. Resonant Frequency

The resonant frequency of a vibration energy harvester is one of the most important design parameters, because the maximum output power can be obtained when the vibration frequency matches the resonant frequency. It is well-known that the power output will be dramatically reduced when the driving vibration frequency deviates from the resonant frequency of a device [16]. When a horizontal cantilever beam is subjected to a vertical harmonic excitation acting at the tip of the beam, the equivalent stiffness is obtained from the following Equation (4):

(4) $$k_{\text{eq}}=\frac{3EI}{L^{3}}$$

where *E* is the equivalent elastic modulus, *I* is the equivalent rational inertia, and *L* is the effective length of the cantilever beam. The value of *EI* can be calculated by Equation (5) [17]:

(5) $$\text{EI}=E_{p1}W_{p}h_{p}\;\left[{\left(z_{pN}-z_{N}\right)}^{2}+\frac{h_{p}^{2}}{12}\right]+E_{s1}W_{s}h_{s}\;\left[{\left(z_{sN}-z_{N}\right)}^{2}+\frac{h_{s}^{2}}{12}\right]$$

where *z_pN_* and *z_sN_* are the neutral axis of the piezoelectric layer and silicon substrate, *z_N_* is the neutral axis of the total piezoelectric cantilever. They are calculated by Equations (6)–(8) respectively:

(6) $$z_{pN}=\frac{1}{2}h_{p}$$

(7) $$z_{sN}=h_{p}+\frac{1}{2}h_{s}$$

(8) $$z_{N}=\frac{E_{p1}h_{p}z_{pN}+E_{s1}h_{s}z_{sN}}{E_{p1}h_{p}+E_{s1}h_{s}}$$

where *E_p1_* and *E_s1_* are the Young's modulus of PZT material and Si beam under the plane strain respectively, they are expressed by Equations (9) and (10) as follows:

(9) $$E_{p1}=\frac{E_{p}}{1-\nu_{p}^{2}}$$

(10) $$E_{s1}=\frac{E_{s}}{1-\nu_{s}^{2}}$$

where *E_p_* and *E_s_* are the elastic modulus of PZT material and Si material respectively, *v_p_* and *v_s_* are the Poisson's ratio of the PZT layer and silicon cantilever beam layer. Based on the equivalent model of the cantilever beam, the resonant frequency of the piezoelectric cantilever beam can be written as a simple Equation (11) [16]:

(11) $$f_{n}=\frac{\omega_{n}}{2\pi }=\frac{1}{2\pi }\sqrt{\frac{3EI}{(m+\frac{33}{140}m_{s})L^{3}}}$$

where *L* is the equivalent length of the beam and the mass, *m* is the mass of the proof mass, and *m_s_* is the mass of the piezoelectric cantilever.

## 3. Finite Element Method Model

**Figure 2.**
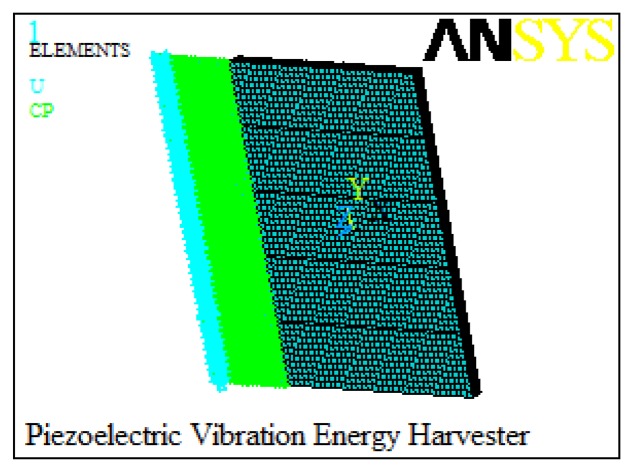
Finite element model.

The proposed piezoelectric energy harvester is analyzed using FEM to predict the relationships between output characteristics and geometries of the harvester. The piezoelectric energy harvester is modeled by the ANSYS14.0 software. Element types of the elastic base and piezoelectric patches are selected as ‘SOLID45’ and ‘SOLID5’, respectively. A mapped meshing method is specified to mesh the created model. In addition, the boundary conditions are applied to the clamped end of the cantilever by applying zero displacement for all degrees of freedom at the nodes. The top and bottom electrodes of the five piezoelectric patches are defined respectively [18]. Figure 2 shows the created finite element model of the piezoelectric energy harvester.

**Figure 3.**
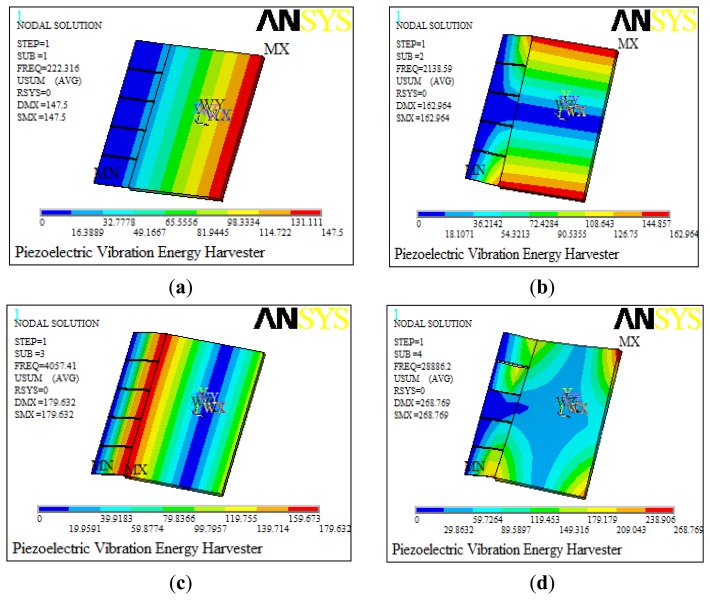
The first four resonant frequencies of the piezoelectric energy harvester. (**a**) The first resonant frequency. (**b**) The second frequency. (**c**) The third resonant frequency. (**d**) The fourth resonant frequency.

A modal analysis is conducted firstly for the established model to determine the vibration modes and corresponding resonance frequencies. The resonance frequencies for the first four models are 222.3 Hz, 2,138.6 Hz, 4,057.4 Hz and 28,886.2 Hz, respectively, simulated by using ANSYS software as shown in Figure 3. The horizontal color bar refers to the modal displacements of each point in the structure, and its unit is meters. However, the modal displacements here are not really physical displacements but the relative displacements, and they illustrate the vibration characteristics of the structure. The first resonant frequency (222.3 Hz) of the model makes it possible to use it in the low frequency range, which is always chosen for dynamic analysis to achieve the maximum power. Besides, there is a quite difference between the first frequency and the others, which ensures the stability of the piezoelectric energy harvester.

**Figure 4.**
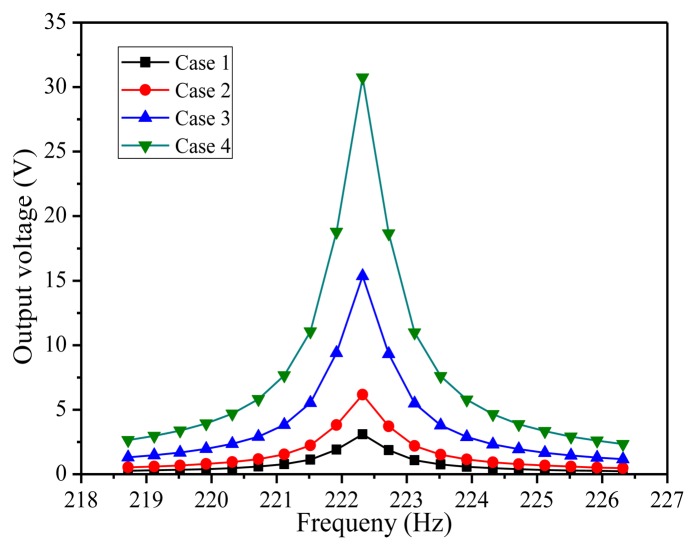
Harmonic curves.

Secondly, a harmonic analysis is implemented to calculate the response of the structure to cyclic loads over a frequency range, which can provide the curves between the induced voltage and the dynamic frequency. This can help the designer evaluate the dynamic traits of the results and any possible fatigue and crack problems to maintain the stability of the device. In the harmonic response analysis process, sinusoidal signal *z*(*t*) = *u*_0_*sin*(*ωt*) is forced on the whole structure in the poling direction of the piezoelectric patch. Figure 4 shows the curves between the induced voltage and the dynamic frequency in four cases. Case 1 and Case 2 show the electric potential of single beam and five series connected beams at 5 m/s^2^ vibration acceleration, respectively. Case 3 and Case 4 present separately the output voltages derived from single beam and five connected beams in series at 10 m/s^2^ vibration acceleration. All four simulation results indicate that maximum electric potential occurred at the first resonance frequency of 222.3 Hz and then declined rapidly. That is to say, the piezoelectric cantilever is only sensitive to the harmonic frequency. If the acceleration is twice as much as before, the value of output voltage is doubled, which is mainly due to the fact that harmonic analysis is a linear analysis. Besides, the output voltage of five beams connected in series happened to be nearly five times as much as that of single beam as a result of the symmetry of the piezoelectric vibration structure. The output voltage response of piezoelectric energy harvester can be seen distinctly under differing frequency exciting forces by the harmonic response, which provides the theory for further systematic dynamic analysis and the structure design of the energy harvester. These also provide an applied dynamic force analysis method, which can be used as reference for other analysis.

**Figure 5.**
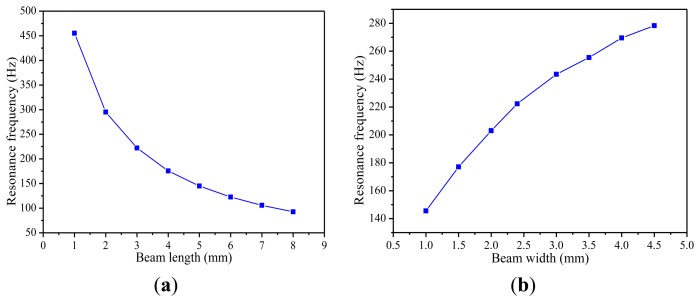

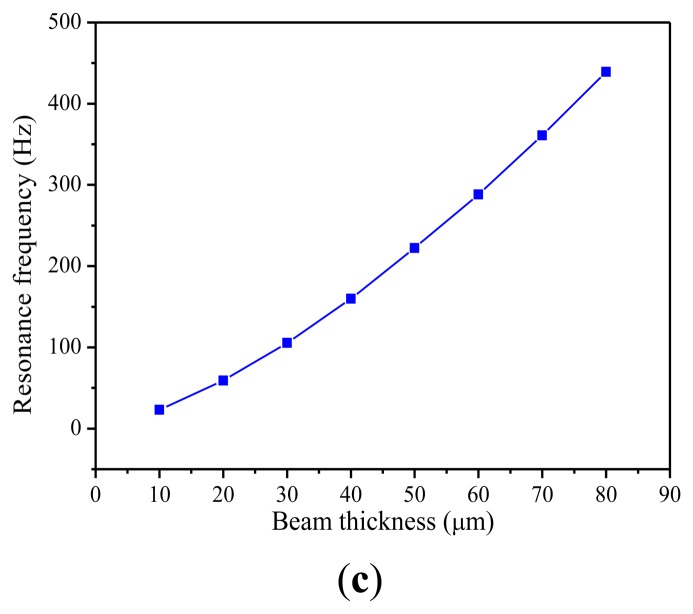
Resonance frequency versus dimensions of the beam. (**a**) Beam length. (**b**) Beam width. (**c**) Beam thickness.

Thirdly, the paper also studies how the resonant frequencies of the piezoelectric energy harvester structure are affected by the length, the width and the thickness of the cantilever beam. Different simulation results are plotted in Figure 5. Figure 5a shows that when the width and thickness of the cantilever beam are fixed, the resonance frequency decreases with the increase of the length of the cantilever beam. As shown in Figure 5b, with the increasing width of the cantilever beam, there is a clear growth trend in the resonance frequency.

In Figure 5c, it can be seen that the resonant frequency increases nearly linearly with the thickness. Accordingly, in order to make the piezoelectric energy harvester work in a low vibration frequency environment, the cantilever beam should be designed to be longer, narrower and thinner on the condition that it cannot be fractured.

**Figure 6.**
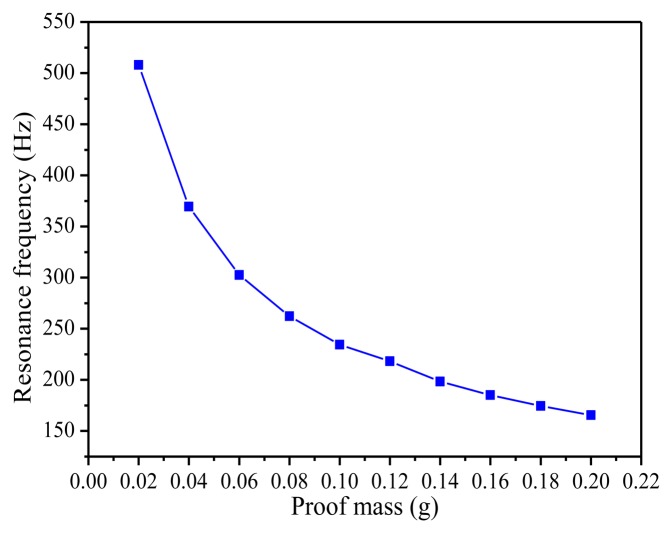
The resonance frequency *versus* the proof mass.

Figure 6 presents the relationship between resonance frequency of the piezoelectric energy harvester and the mass at the tip of the beam. The resonance frequency decreases sharply from 508.0 Hz to 165.53 Hz when the mass of the proof mass increases from 0.02 g to 0.2 g. As a result, by adjusting the mass of the proof mass, the resonance frequency of the piezoelectric energy harvester can be significantly changed to make it work better to meet the low frequency vibration needs.

It is obvious that the targeted resonant frequency is attained by changing the lengths and widths of the cantilever beam and the dimensions of the proof mass. Based on the simulation results, the structural parameters of the piezoelectric energy harvester in Table 1 are chosen in this design. Substituting the involved parameters into Equation (5), the value of *EI* is as follows: *EI* = 4.95 × 10^−6^. Then, substituting the value of *EI*, *L*, *m*, *m_b_* into Equation (11), the theoretical value of resonance frequency is as shown in Equation (12). Compared the theoretical value of resonance frequency with the simulation resonance frequency of Figure 4, the relative error value between theoretical and simulation resonant frequency is only 2%. This result shows the theoretical analysis has high prediction accuracy, which provides the basis of architecture parameters design for MEMS PZT energy harvester:

(12) $$f_{n}=\frac{\omega_{n}}{2\pi }=\frac{1}{2\pi }\sqrt{\frac{3EI}{(m+\frac{33}{140}m_{s})L^{3}}}=217.65\;(Hz)$$

**Figure 7.**
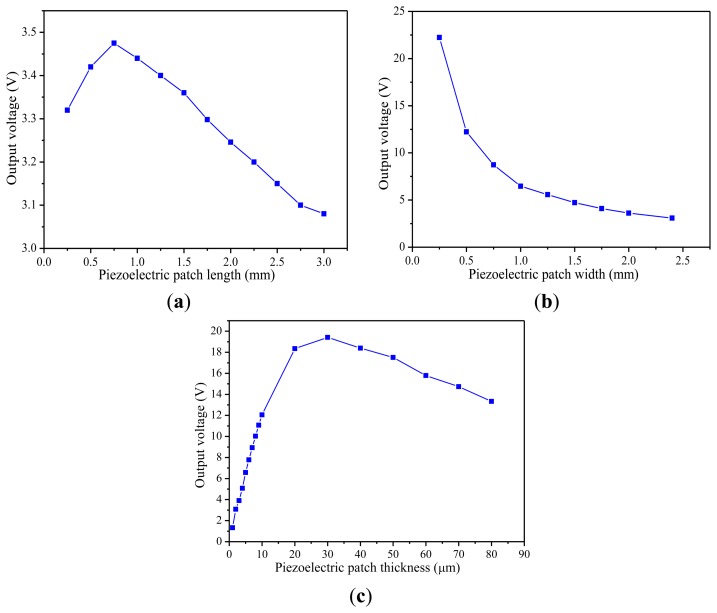
Output voltage versus piezoelectric patch dimensions. (**a**) Patch length. (**b**) Patch width. (**c**) Patch thickness.

Finally, to investigate the dependence of the output voltage on the parameters of the piezoelectric patch, additional harmonic analyses are done. Three plots of the relationships between them are shown in Figure 7. Figure 7a shows the dependence of the output voltage on the length of the piezoelectric patch. While other parameters remain constant, the output voltage first increases and then decreases with the increase of the length of piezoelectric patch. From Figure 7b, it is found that the output voltage decreases when the width of the piezoelectric patch is changed from 0.25 mm to 2.4 mm. The result in Figure 7c shows that the output voltage begins to increase first, and then decreases with the growth of the thickness of the piezoelectric patch. The analysis results show that the piezoelectric patch with the appropriate length and thickness should be less wide compared with the dimensions of substrate, which is well in agreement with the previous literatures [19].

Based on these simulation results, a MEMS piezoelectric cantilevers array (five beams of 3 × 2.4 × 0.05 mm^3^) integrated with a large silicon proof mass of about 8 × 12.4 × 0.5 mm^3^ is designed to improve the output voltage for the low frequency vibration condition.

## 4. Fabrication

**Figure 8.**
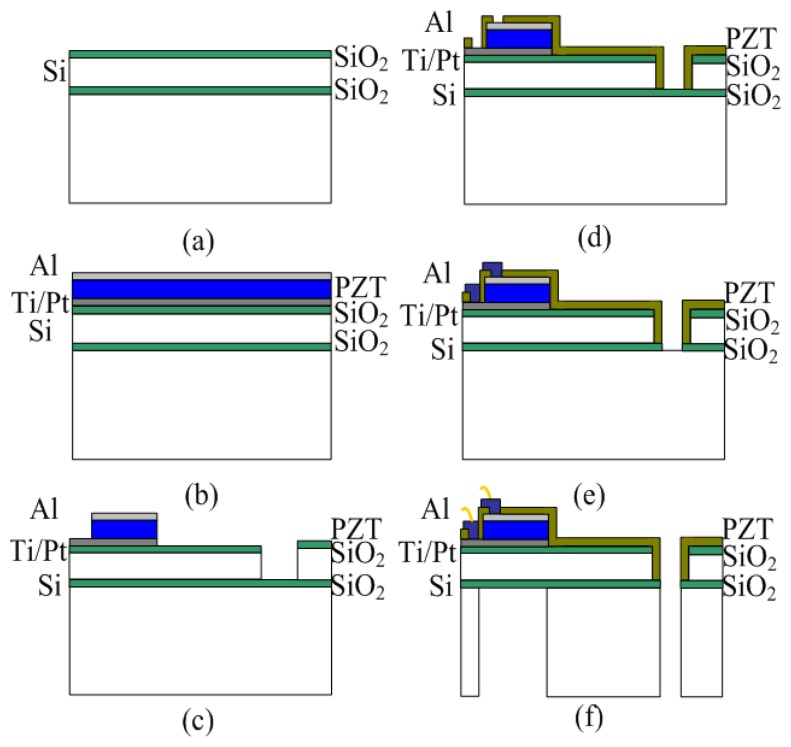
Fabrication process of MEMS piezoelectric energy harvester.

The MEMS piezoelectric energy harvester consists of five piezoelectric PZT cantilevers and an integrated silicon proof mass. The five PZT elements are electrically isolated from one another and each PZT element is composed of a top electrode layer (Al), a PZT layer, and a bottom electrode layer (Pt/Ti). Each of the top and bottom electrodes of the PZT element are connected to a bonding pad individually. A large proof mass is designed and integrated at the end of the supporting beam to achieve a low resonant frequency and increase output power. The fabrication process is as shown in Figure 8 [20,21]:

(a) Grow thermal oxide on silicon on insulator (SOI) wafer.
(b) Sputter deposit Ti/Pt (bottom electrode), and *Al* (top electrode), spin coat sol-gel PZT piezoelectric layer.
(c) Pattern and etch electrode, PZT and SiO_2_ layer, deep reactive ion etching (DRIE) Si device layer.
(d) Plasma enhanced chemical vapor deposition (PECVD) deposit oxide, pattern and etch via.
(e) Pattern and etch contact pads.
(f) DRIE from backside, dicing and releasing.

## 5. Power Conditioning Circuit

**Figure 9.**
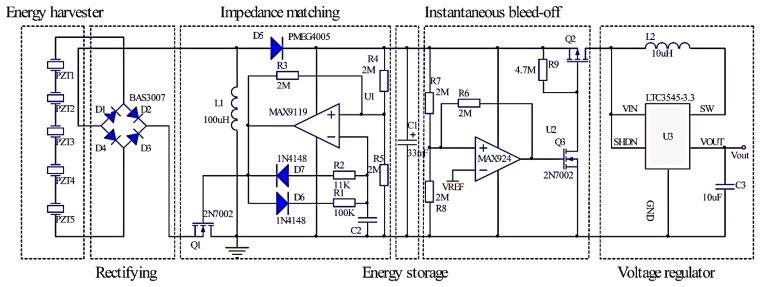
Power conditioning circuit for PZT energy harvester.

The main goal of the power conditioning circuit is highly efficient energy transfer and energy accumulation between the harvester and the electric load, because the typical output power of a MEMS vibration energy harvester is in the μW range. Figure 9 shows the schematic of the proposed power conditioning circuit, which includes AC-DC rectifying, impedance matching, energy storage, instantaneous bleed-off and voltage regulator circuits. The basic design idea is to enable maximum power extraction from the piezoelectric energy harvester by using the impedance matching circuit, store the energy in a super-capacitor and supply power to the load when enough energy is accumulated in super-capacitor. The different parts of the circuit are analyzed with detailed information as follows.

### 5.1. Rectifying Circuit

The output of the piezoelectric energy harvester is an alternating voltage signal which must be rectified for supplying power to the sensor node load. The rectifying bridge circuit consists of four small-signal diodes. These diodes are chosen specifically for rectification because they must have the smallest forward voltage drop and leakage current.

### 5.2. Impedance Matching Circuit

The discontinuous control mode (DCM) buck-boost converter is chosen for the second stage because of its ability to accommodate a wide range of input voltage and behave as a lossless resistor to match the source impedance for the maximum power point tracking. The effective input resistance of a DCM buck-boost converter is given in Equation (13) as follows [22]:

(13) $$R_{IN}=\frac{V_{\text{RECT}}}{\frac{1}{T_{S}}\int_{0}^{D_{1}T_{S}}I_{L1}dt}=\frac{2L_{1}}{D_{1}^{2}T_{S}}$$

where *T_s_* is switching period of the transistor *Q*_1_, *D*_1_ is duty cycle of the transistor *Q*_1_, and *V_RECT_* is the rectified voltage. In order to achieve the resistive impedance matching, the effective input resistance *R_IN_* should be equal to the optimal resistive load *R_L,opt_*. Hence, the optimal duty cycle can be expressed by using Equation (14):

(14) $$D_{1,\text{OPT}}=\sqrt{\frac{2L_{1}}{R_{\text{IN},\text{OPT}}T_{S}}}$$

An ultra-low power oscillator with an *RC* network is used to generate the pulse width modulation (PWM) signal for turning the power switch *Q*_1_ on or off. The duty cycle and switching frequency can be adjusted by choosing appropriate *R_1_*, *R_2_* and *C_2_* to achieve the maximum power delivery in Figure 9. If the comparator output is high, the voltage at the non-inverting input of the comparator is two-thirds of the supply voltage. Capacitor *C_2_* is charged through resistor *R_1_*. As soon as the capacitor voltage reaches two-thirds of the supply voltage, the comparator output acts and goes low. The voltage at the non-inverting input of comparator is one-third of the supply voltage. Capacitor *C_2_* is discharged through resistor *R_2_*. The power supply of the chip connects to the cathode of diode *D_5_*.Once the capacitor voltage decreases one-third of the supply voltage, the comparator output becomes high, Capacitor *C_2_* is charged again and the whole cycle repeats.

### 5.3. Energy Storage and Instantaneous Bleed-Off Circuit

In order to drive the load successfully, the weak harvested energy should be accumulated in the energy storage element during the long period time because a wireless sensor node requires much more peak power than a piezoelectric micro power generator can produce. A super-capacitor *C_1_* of 33 mF is chosen as a storage element in this energy harvesting system, which accumulates energy to enable efficient use for short power output bursts. The super-capacitor should be disconnected from the load during the energy accumulation stage to prevent energy leakage to the load, and is supposed to be connected to the load only if the accumulated energy is large enough to drive it. The instantaneous bleed-off circuit is composed of an ultra-low power voltage comparator *U_2_* with hysteresis, the switching *Q*_2_, and *Q*_3_. The comparator monitors the super-capacitor's voltage and controls the super-capacitor's charging and discharging process. The switch is in charge of powering on or off the wireless sensor node. The hysteresis of a comparator creates two threshold voltage points: the upper one for super-capacitor charge voltage and the other lower one for the super-capacitor discharge voltage, which can control how much energy discharging from the super-capacitor.

### 5.4. Voltage Regulator

A voltage regulator is adopted as the output unit providing a stable voltage supply to the wireless sensor node. In order to improve the efficiency of the voltage regulator, two key design points are applied in the circuit. One is to reduce inductor power loss and the other is set to enable start-up signal for DC-DC converter. If input voltage of the voltage regulator (*i.e.*, the super-capacitor's voltage) is lower than a defined start-up voltage, the voltage regulator consumes unnecessary power because it is not able to boost the output voltage. Thus, to overcome this drawback, we introduce a supervisory unit that continuously checks the super-capacitor's voltage and enables the voltage regulator output stage only when it can be equal to the value of startup voltage. Otherwise, a complete shutdown with output disconnection suppresses any additional power consumption, reducing the charging time of the super-capacitor and boosting the overall efficiency.

## 6. Testing and Analysis

### 6.1. Experimental Test Setup

**Figure 10.**
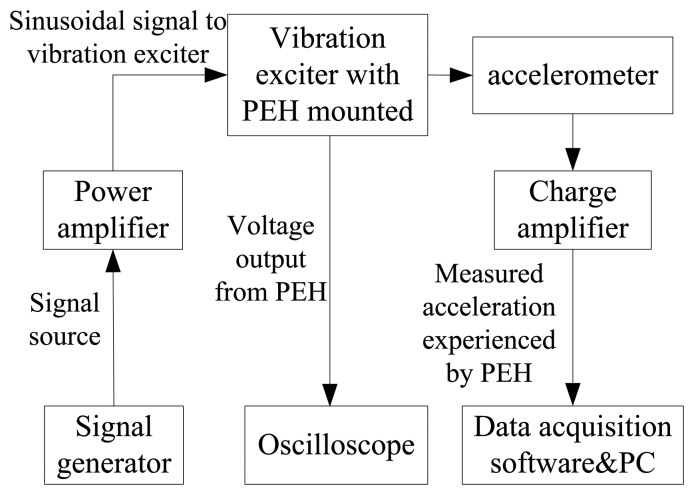
Schematic drawing of the experimental setup for PEH system.

**Figure 11.**
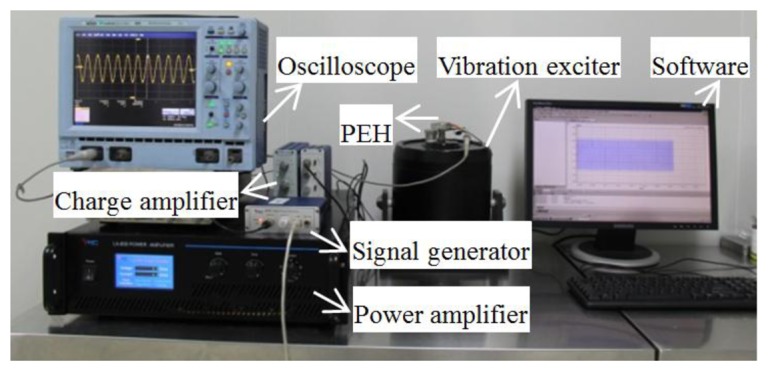
Photo of the experimental setup for MEMS piezoelectric energy harvester.

In order to measure the output characteristics of the piezoelectric energy harvester, a vibration testing system is employed. A schematic drawing of the experimental setup for PEH system is shown in Figure 10. It consists of a vibration exciter, an accelerometer, a power amplifier, an oscilloscope, a signal generator and a charge amplifier. An accelerometer is assembled on the vibration exciter together with the cantilever array device for the acceleration measurement. The vibration signal is generated from the signal generator, amplified via the power amplifier and finally utilized to control the vibration amplitude and frequency of the shaker. Accordingly, the piezoelectric cantilever device will undergo excitations and generate output voltage signal which is recorded to the oscilloscope. Acceleration signals will be measured by the accelerometer and amplified by the charge amplifier, and then shown on the monitor of the computer. The photo of the experimental system for testing the MEMS piezoelectric energy harvester is shown in Figure 11.

### 6.2. Test Results and Analysis

**Figure 12.**
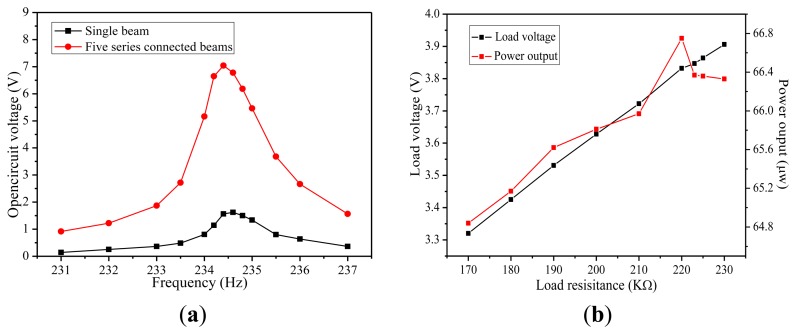
The characteristics of MEMS piezoelectric energy harvester. (**a**) Open circuit voltage *vs*. the frequency. (**b**) Load voltage and power *vs*. the load.

First, the respective characteristics of the MEMS piezoelectric energy harvester are measured, including the resonant frequency, output voltage, output power and optimal load. Figure 12a shows the output voltage of a single PZT beam and five beams connected in series as the vibration frequency swept from 231 Hz to 237 Hz at 5 m/s^2^ vibration acceleration. As can be seen, the maximum open circuit voltage is 1.62 V and 7.04 V at the resonant frequency of 234.5 Hz under 5 m/s^2^ acceleration, respectively. The error between the simulated and measured resonant frequency is about 4.2%. Figure 12b shows the load voltage and average power to the different loads at resonant frequency. As expected, the load voltage increases with the increased load, up to 3.91 V at 230 KΩ load. However, the power delivered to the load has a maximum value. The maximum output power of 66.75 μW is obtained at 5 m/s^2^ acceleration with an optimal resistive load of 220 kΩ resulting in a maximum power density of 5.19 μW·mm^−3^·g^−2^. The power density is calculated using the average power divided by the effective volume of the device, which is the volume of the entire beam and the Si proof mass calculated from the measured data in Table 1. Some main performance parameters of recent MEMS piezoelectric energy harvesters are compared in Table 2.

**Figure 13.**
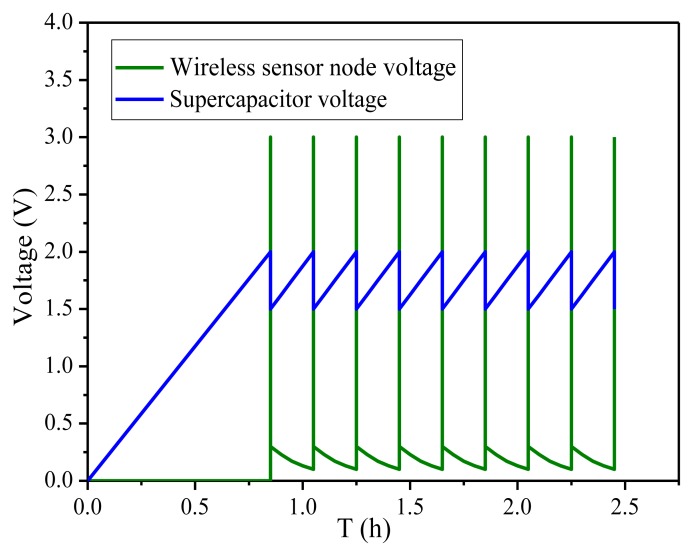
The voltage across the super-capacitor and wireless sensor node.

The next experiment shows the performance of the power conditioning circuit. Figure 12 shows that the proposed power MEMS system can successfully drive the wireless sensor load when the temperature-humidity sensor node transmits signals. As shown in Figure 13, when the voltage across the super-capacitor is charged to 2 V, the instantaneous bleed-off circuit begins to work and the voltage regulator will start up, supplying 3.3 V to the wireless sensor node load. The super-capacitor's voltage will begin to drop while the energy is supplied to the load. When the voltage across the super-capacitor drops below 1.5 V, the output voltage level of instantaneous bleed-off circuit will become low, so the voltage regulator shut off again. When the super-capacitor voltage is charged to 2.0 V next time, the DC-DC converter will start up again. Through the test, the working cycle time of the wireless sensor node is 0.185 h. In the proposed circuit, the energy of super-capacitor discharging is shown in Equation (15) and the efficiency of the energy management is calculated by Equation (16), respectively:

(15) $${\text{E}}_{\text{SC}}=\frac{1}{2}{\text{CV}}_{\text{THR}}^{2}-\frac{1}{2}{\text{CV}}_{\text{THF}}^{2}=\frac{1}{2}\times 33\text{mF}\times 10^{-3}\times \left(2^{2}-1.5^{2}\right)\text{V}=28.875\text{mJ}$$

(16) $$\eta =\frac{P_{\text{stored},SC}}{P_{\text{MAP}}}=\frac{E_{SC}/T}{P_{\text{MAP}}}=\frac{28.875mJ/0.185h}{66.75\mu W}=64.95\%$$

The maximum available power of 66.75 μW is measured as the power delivered to the 220 kΩ optimal resistive load connected directly to the piezoelectric generator at the resonant frequency of 234.5 Hz. The extracted power of the proposed power conditioning circuit is the power delivered to the super-capacitor during the charging process. The proposed circuit's efficiency is about 64.95%, which is better than the results reported in some previous literature [27–29].

## 7. Results

In this paper, a self-supplied MEMS piezoelectric energy generator with power conditioning circuit is proposed. A complete design flow analyzing the architecture and parameters of the energy harvester using the FEM is established. Based on the simulation results, a MEMS PZT cantilever array with an integrated large Si proof mass is designed and fabricated. Test results show that the proposed energy harvester can produce a maximum output power of 66.75 μW, or power density of 5.19 μW·mm^−3^·g^−2^ with an optimal resistive load of 220 kΩ from 5 m/s^2^ acceleration at its resonant frequency of 234.5 Hz, which represents a significant improvement of output power and power density. This paper also offer a design method of a power conditioning circuit with the function of impedance matching, energy storage and voltage regulation in order to improve high impedance characteristics of PZT energy harvester and attain higher efficiency. The experimental results show that the proposed self-supplied energy generator with power conditioning circuit could provide a more promising complete power supply solution for wireless sensor node loads. Future planned work includes further improvement of the energy harvester architecture and development of a monolithic power conditioning chip.

## Figures and Tables

**Table 1.** Material properties and structural parameters of the piezoelectric energy harvester.

**Symbol**	**Parameter**	**Value**
*ρ_S_*	Density of Si beam	2,329 kg/m^3^
*ρ_P_*	Density of PZT	7,500 kg/m^3^
*E_S_*	Young's modulus of Si beam	170 GPa
*E_P_*	Young's modulus of PZT	98.4 GPa
*ν_S_*	Poisson's ratio of Si beam	0.28
*ν_P_*	Poisson's ratio of PZT	0.29
*d_31_*	Piezoelectric constant of PZT	−274 × 10^−12^ C/N
*ε*_0_	Vacuum dielectric constant	8.85 × 10^−12^ F/M
$\varepsilon_{11}^{s}/\varepsilon_{0}$	Relative dielectric constant	1,703
$\varepsilon_{33}^{s}/\varepsilon_{0}$	Relative dielectric constant	1,433.6
*l_S_* × *w_S_* × *h_S_*	Dimensions of Si beam	3 × 2.4 × 0.05 mm^3^
*l_P_* × *w_P_* × *h_P_*	Dimensions of PZT	3 × 2.4 × 0.002 mm^3^
*w_g_*	Gaps between the beams	100 μm
*l_m_*× *w_m_* × *h_m_*	Dimensions of the proof mass	8 × 12.4 × 0.5 mm^3^

**Table 2.** Comparison of recent MEMS piezoelectric energy harvesters.

**Reference**	**Material**	**Volume mm^3^**	**A m/s^2^**	**f Hz**	**Power μW**	**Power Density** **μW·mm^−3^·g^−2^**
[23]	PZT *d_31_*	1.063	5	243	2.33	8.39
[24]	PZT *d_31_*	12.775	9.3	615	51.3	4.97
[25]	PZT *d_31_*	27	15	154	205	3.37
[26]	PZT *d_31_*	0.608*	10	78.7	0.803	1.32
This paper	PZT *d_31_*	51.472	5	234.5	66.75	5.19

* Estimated from reference data.
